# Current Research and Opportunities to Address Environmental Asbestos Exposures

**DOI:** 10.1289/ehp.1409662

**Published:** 2015-08-01

**Authors:** Danielle J. Carlin, Theodore C. Larson, Jean C. Pfau, Stephen H. Gavett, Arti Shukla, Aubrey Miller, Ronald Hines

**Affiliations:** 1 Division of Extramural Research and Training, National Institute of Environmental Health Sciences (NIEHS), National Institutes of Health (NIH), Department of Health and Human Services (DHHS), Research Triangle Park, North Carolina, USA; 2 Division of Toxicology and Human Health Sciences, Agency for Toxic Substances and Disease Registry (ATSDR), Atlanta, Georgia, USA; 3 Department of Biological Sciences, Idaho State University, Pocatello, Idaho, USA; 4 Environmental Public Health Division, National Health and Environmental Effects Research Laboratory, U.S. Environmental Protection Agency (EPA), Research Triangle Park, North Carolina, USA; 5 Department of Pathology and Laboratory Medicine, University of Vermont, College of Medicine, Burlington, Vermont, USA; 6 Office of the Director, NIEHS, NIH, DHHS, Bethesda, Maryland, USA; 7 Environmental Public Health Division, National Health and Environmental Effects Research Laboratory, U.S. EPA, Research Triangle Park, North Carolina, USA

## Abstract

Asbestos-related diseases continue to result in approximately 120,000 deaths every year in the United States and worldwide. Although extensive research has been conducted on health effects of occupational exposures to asbestos, many issues related to environmental asbestos exposures remain unresolved. For example, environmental asbestos exposures associated with a former mine in Libby, Montana, have resulted in high rates of nonoccupational asbestos-related disease. Additionally, other areas with naturally occurring asbestos deposits near communities in the United States and overseas are undergoing investigations to assess exposures and potential health risks. Some of the latest public health, epidemiological, and basic research findings were presented at a workshop on asbestos at the 2014 annual meeting of the Society of Toxicology in Phoenix, Arizona. The following focus areas were discussed: *a*) mechanisms resulting in fibrosis and/or tumor development; *b*) relative toxicity of different forms of asbestos and other hazardous elongated mineral particles (EMPs); *c*) proper dose metrics (e.g., mass, fiber number, or surface area of fibers) when interpreting asbestos toxicity; *d*) asbestos exposure to susceptible populations; and *e*) using toxicological findings for risk assessment and remediation efforts. The workshop also featured asbestos research supported by the National Institute of Environmental Health Sciences, the Agency for Toxic Substances and Disease Registry, and the U.S. Environmental Protection Agency. Better protection of individuals from asbestos-related health effects will require stimulation of new multidisciplinary research to further our understanding of what constitutes hazardous exposures and risk factors associated with toxicity of asbestos and other hazardous EMPs (e.g., nanomaterials).

## Background

Exposures to asbestos and similar elongated mineral particles (EMPs) often result in diseases such as pleural plaques, lung cancer, and mesothelioma, which have resulted in approximately 120,000 deaths every year in the United States and worldwide (WHO 2014). Asbestos and EMPs have also been associated with noncancerous diseases such as autoimmune diseases (Pfau et al. 2014). Moreover, these diseases often have long latency periods––making the diagnosis of the disease difficult and associating the illness with the specific exposure challenging. Most of what is known about the health effects associated with asbestos exposure has been due to extensive research on occupational exposures to asbestos (NIOSH 2011), but many issues related to environmental asbestos exposures still remain unresolved. For example, a well-recognized example of environmental asbestos exposure is the town of Libby, Montana, where high rates of nonoccupational asbestos-related diseases have been associated with a former vermiculite mining operation (U.S. EPA 2014b). Other potential environmental exposures are also undergoing investigations to assess exposures and potential health risks: These include naturally occurring asbestos and other EMP deposits in the United States such as tremolite in El Dorado Hills, California (ATSDR 2015; U.S. EPA 2014d); chrysotile in Nooksack and Sumas, Washington (U.S. EPA 2014d); erionite in North Dakota (Carbone et al. 2011); and amphiboles and erionite in Southern Nevada (Baumann et al. 2015). Outside the United States, investigations include crocidolite in the Wittenoom mine in Western Australia (de Klerk et al. 2013); erionite in Sivas province in Turkey (Carbone et al. 2011); and, more recently, erionite in Central Mexico (Ortega-Guerrero et al. 2015). The importance of environmental exposure to asbestos and EMPs is demonstrated in more than 600 reviews (e.g., Norbet et al. 2015; Boulanger et al. 2014), recent commentaries (e.g., Haynes 2010; LaDou et al. 2010), and meetings (e.g., Gwinn et al. 2011). The overarching conclusion in the literature is that the toxicity of occupational asbestos materials has been well characterized (e.g., chrysotile and crocidolite), but more research is needed to determine the relative toxicity of environmental asbestos and EMPs (e.g., erionite and nanomaterials).

## Presentation Topics

A workshop titled “New Concerns and New Science Addressing Environmental Asbestos Exposures” was presented at the 2014 meeting of the Society of Toxicology. The presenters—scientists from both federal agencies [i.e., National Institute of Environmental Health Sciences (NIEHS), U.S. Environmental Protection Agency (EPA), and Agency for Toxic Substances and Disease Registry (ATSDR)] and universities (http://ntp.niehs.nih.gov/pubhealth/evalatm/publications-and-presentations/presentations-at-scientific-meetings/sot-2014/index.html)—highlighted some of the latest findings and recommendations for future research directions related to environmental asbestos exposures (i.e., end-users of asbestos-containing materials, family members of exposed workers, and those living or working in/around contaminated buildings or areas). Specific topics included discussion of the research on the public health situation in Libby, Montana, asbestos and autoimmunity, critical factors for determining asbestos-associated pathologies (e.g., fiber chemistry, size characteristics, and dose), and the role of the inflammasome in asbestos-related disease (ARD).

Despite recent progress, more research is needed to further our understanding of the toxicity and risk factors associated with asbestos and other hazardous elongated particles. Studies that focus on emerging naturally occurring EMPs (some of which are just being discovered) and carbon nanotubes and nanofibers are required to better assess relevant exposures and institute appropriate public health protection.

## Discussion

For the past decade, considerable research has focused on asbestos contamination and high levels of ARD among residents of Libby, Montana. Libby, which was declared a public health emergency in 2009 (U.S. EPA 2014c), was the site of a former mine that produced vermiculite contaminated with a mixture of asbestiform amphiboles, including winchite, richterite, and tremolite asbestos (Meeker et al. 2003). Studies of this population have shown:

Elevated levels of ARD among residents without occupational exposures (Peipins et al. 2003), including reports of atypical pleural abnormalities and elevated respiratory symptoms among those exposed during childhood (Vinikoor et al. 2010).
Potentially shorter latencies of pleural disease among Libby amphibole (LA)–exposed workers compared with workers exposed to other forms of asbestos (Larson et al. 2010).
More frequent and severe pleuritic pain, and rapid loss of pulmonary function compared with reported observations of populations exposed to other forms of absestos (American Thoracic Society 2004; Whitehouse et al. 2008; Black et al. 2014).
Elevated rates of self-reported systemic autoimmune disease (i.e., scleroderma, lupus, and rheumatoid arthritis) (Noonan et al. 2006).
Findings of higher prevalence of positive antinuclear antibody and extractable nuclear antigen test results compared with an age- and sex-matched population from a region of similar geography and meteorology but with no known asbestos exposure (Pfau et al. 2005).

In related studies, the prevalence of pleural plaques was increased among workers in Marysville, Ohio, who had very low lifetime cumulative fiber exposures from processing Libby vermiculite; these pleural changes were also associated with spirometric decrements (Lockey et al. 1984; Rohs et al. 2008; Lockey et al. 2015). Additionally, an extensive toxicologic review and risk assessment recently released by the U.S. EPA found noncancerous pleural disease to be the most sensitive health effect at the Libby site rather than mesothelioma and lung cancer—both longstanding sensitive health endpoints for asbestos risk assessments (U.S. EPA 2014b).

Currently, two research programs are underway to further investigate the health effects associated with the LA exposures:
The University of Cincinnati Childhood Health Investigation and Exposure Follow-up Study. This health study of Libby residents who were children when the mine was opened included medical examinations, radiological tests and pulmonary function testing, as well as reconstructing a history of childhood exposures (Ryan et al. 2015).
The Icahn School of Medicine at Mount Sinai Libby Epidemiology Research Program. This project has three objectives: examine pulmonary disease progression using high-resolution computed tomography; evaluate pulmonary health of former child residents (i.e., high-school graduates who have moved away from Libby); and investigate the relationship between residential exposure, autoimmunity, and ARD (Mount Sinai Hospital 2009).

Another topic at the workshop was immune dysfunction as a part of the response following asbestos exposure. Several reports indicate increased autoantibodies such as rheumatoid factor and anti-nuclear autoantibodies (ANA) in asbestos-exposed populations (Pfau et al. 2014). However, epidemiological data that clearly links asbestos exposure with clinically diagnosed autoimmune disease is limited––just a handful of studies have shown an association of asbestos exposure and rheumatoid arthritis and systemic sclerosis (reviewed in Pfau et al. 2014)––and a few studies have identified an increased risk of systemic autoimmune diseases among persons with known asbestos exposures (i.e., Libby, Montana; Pfau et al. 2005; Noonan et al. 2006).

Several studies have reported evidence that asbestos disease outcomes may be critically affected by the immunological impacts of specific fiber types. C57BL/6 mouse studies have shown evidence that LA material increases the risk of autoimmune responses including autoantibodies and Th17 cytokines detected in serum (Ferro et al. 2014). Interestingly, erionite, a hazardous zeolite EMP, also induced a similar set of responses in this same strain of mouse (Zebedeo et al. 2014). However, chrysotile did not have this effect; instead, it showed a somewhat immunosuppressed serum cytokine profile (Zebedeo et al. 2014). Overall, the findings suggest that fiber type, as well as other fiber morphologic characteristics, must be considered when exploring the immune and other health effects of asbestos and asbestos-like EMPs. The lack of studies comparing autoimmune responses among populations exposed to different types of fibers may be one of the reasons why there is a lack of clear epidemiological association between “asbestos” and systemic autoimmune diseases (Pfau et al. 2014).

Autoantibodies to fibroblasts (AFA) have also been implicated in fibrotic diseases such as systemic sclerosis (Chizzolini et al. 2002). Amphibole-exposed mice have been shown to produce AFA, which induces collagen production and a profibrotic phenotype (Pfau et al., 2011). LA exposure also induces production of autoantibodies to mesothelial cells (Marchand et al. 2012). These antimesothelial cell autoantibodies induce collagen production from human mesothelial cells in culture (Serve et al. 2013). These studies, along with epidemiologic evidence of high rates and unusual clinical manifestation of pleural disease among Libby residents, suggest that further investigation is needed to assess the possibility of an autoimmune contribution to pleural disease.

The workshop also included discussion of some of the determinants of toxicity of environmental asbestos and EMPs, such as fiber chemistry, length, aspect ratio, surface area, dose, biopersistence, and underlying disease (e.g., cardiovascular disease; Shannahan et al. 2011a, 2011b, 2012). Studies that were highlighted used respirable samples (i.e., aerodynamic diameter ≤ 2.5 μm) of LA and a sample of a long fiber amosite. The amosite had median lengths about twice that of LA, but the widths of LA and the amosite were equivalent. For the *in vitro* studies, Duncan et al. (2010) found that inflammatory mediators [i.e., interleukin-8 (IL-8) and cyclooxygenase-2 (COX-2)] were 4-fold and 10-fold greater for amosite than for LA, respectively; amosite exposure increased the expression of genes in inflammation pathways, but decreased the expression of genes in oxidative and heat shock pathways. In a more recent study, Duncan et al. (2014) reported that the fiber surface area predicted inflammatory responses of multiple fiber samples more accurately than did fiber number or fiber mass. For the *in vivo* studies (Padilla-Carlin et al. 2011; Cyphert et al. 2012a, 2015), investigators found that intratracheal (IT) exposure of rats to LA and other fibers demonstrated that bronchoalveolar lavage protein, a marker of lung injury, correlated strongly with the number of fibers with lengths of 5–10 μm but not with those longer than 20 μm. This finding could be due to a much smaller fraction of the longer fibers relative to shorter fibers in the LA sample. Lung fibrosis continued to increase in the asbestos-exposed rats: Amosite had the greatest effect compared with the effect of other fibers 2 years after exposure (Cyphert et al. 2012b, 2015).

Comparative toxicology studies of LA with other naturally occurring forms of asbestos were also conducted by Cyphert et al. (2012b) using samples of chrysotile asbestos sediments from a slow-moving landslide on Sumas Mountain, Washington, and from naturally occurring tremolite in El Dorado Hills, California––both areas are of concern due to exposures to local communities. A sample of ferroactinolite cleavage fragments from Ontario, Canada, was also tested on rat lung tissue. Indices of toxicity showed significant effects of Sumas Mountain chrysotile, suggesting concern for the population exposed to materials from this slow-moving landslide.

The need for improved understanding of the mechanisms of asbestos-related disease was also emphasized. For example, inflammasomes are special components of inflammation represented by cytosolic sensors called nucleotide binding and oligomerization domain (NOD)-like receptors (NLRs) (Martinon et al. 2002). In response to various pathogenic and nonpathogenic stressors, these NLRs are primed and subsequently activated. The activation results in production of active caspase-1 that can induce the production of mature IL-1β and IL-18, and thus create a proinflammatory environment. The Nlrp3 inflammasome has been shown to be activated by particles and fibers (Dostert et al. 2008). Four exciting areas of inflammasome research were presented:

The indication that asbestos and erionite exposure can prime and activate Nlrp3 in mesothelial cells (Hillegass et al. 2013).
The role of reactive oxygen species (ROS) in asbestos-induced inflammasome regulation (Thompson et al. 2014).
How the mesothelial cell’s ability to phagocytize asbestos is known to activate the Nlrp3 inflammasome.
How asbestos is involved in the transformation of mesothelial cells and malignant mesothelioma development through the mesothelial to fibroblastic transition process.

The workshop concluded by identifying several challenges and recommendations for future research:

Chemical and physical characterization. Ongoing controversy exists with respect to the potency of various forms of asbestos (i.e., crocidolite, anthophyllite, tremolite, actinolite, amosite, chrysotile). Furthermore, other mineral fibers, not used for commercial purposes or classified as “asbestos” (i.e., magnesio-riebeckite, magnesio-arfvedsonite, winchite, richterite, fluoro-edenite, antigorite, and erionite) are known to be associated with ARD among exposed populations, and health investigations are urgently needed for populations exposed to these mineral fibers. For example, erionite (a zeolite) has resulted in 30–50% of adult mesothelioma deaths in Turkish villages. Erionite has also been found on North Dakota roads (Carbone et al. 2011) and identified in other locations in the United States (Van Gosen et al. 2013). These studies highlight the critical importance for researchers to determine the physical and chemical characteristics that induce adverse health effects so that surveillance of exposed populations and protective measures can be implemented to reduce worker and community exposures.
Regulatory concerns. Asbestos regulations were first developed more than 30 years ago for the workplace (i.e., asbestos product manufacturing) and have primarily relied on phase contrast microscopic (PCM) methods (which quantify fibers > 5 µm in length and > 0.25 µm in width) to identify the presence of asbestos fibers in asbestos-containing materials or in the air (OSHA 1994; Stayner et al. 1997). However, today’s environmental assessments require the use of high-power magnification [e.g., transmission electron microscopy (TEM)] to discern asbestos fibers not counted by PCM approaches (i.e., missing short fibers < 5 µm long and thin fibers < 0.25 µm in diameter), yet some of these noncounted fibers may be toxic (Dement et al. 2015). Additionally, more sensitive analytical techniques will be needed to address materials with asbestos concentrations < 1% by weight (e.g., soils, attic vermiculite) that can still generate hazardous exposures when disturbed (Ewing et al. 2010).
Susceptible populations. Glaring deficiencies exist in the historic strategies used to evaluate nonoccupational asbestos exposures and the risks of ARD in sensitive populations such as children, pregnant women, or those with preexisting disease. For example, children living and playing around the Wittenoom crocidolite mine in Western Australia developed excess rates of brain, ovarian, prostate, and colorectal cancers as adults in addition to mesothelioma (Reid et al. 2013). Thus, children and others who handle asbestos at early life stages could be at increased risk for ARD and other chronic diseases.
A multidisciplinary approach. Research teams that include epidemiologists, toxicologists, mineralogists, clinicians, and statisticians have been working on complex issues such as the Libby, Montana, site and other locations around the United States that contain hazardous mineral fibers. Utilizing interagency working groups and workshops such as the NIEHS-sponsored “Mechanisms of Action” workshop in December 2009 (Chapel Hill, North Carolina), experts have identified data gaps and research needs (Gwinn et al. 2011). The NIEHS National Toxicology Program (NTP) also designs projects (e.g., 2-year bioassays) to better assess the toxicity of LA material in conjunction with a comprehensive program to study naturally occurring asbestos and related mineral fibers (NTP 2007). The NIEHS Superfund Research Program has also recently added the University of Pennsylvania Superfund Center into its grant portfolio (Superfund Research Program 2014). This interdisciplinary center is evaluating the health effects associated with chrysotile found at the Ambler, Pennsylvania, Superfund site (U.S. EPA 2014a). Together, these studies will collect toxicity data, complete detailed physical and chemical characterizations, and develop remediation strategies.

## Conclusions

Although much literature on the topic of asbestos already exists, the 2014 Society of Toxicology workshop indicates that there are new lines of research related to the human health impacts of asbestos that are being actively pursued and that additional questions remain to be addressed (http://ntp.niehs.nih.gov/pubhealth/evalatm/publications-and-presentations/presentations-at-scientific-meetings/sot-2014/index.html). For example, studies of the Libby population, similar to Wittenoom, Australia, and Sivas province in Turkey, will yield additional information helpful to residents and the international scientific community. In addition, attention to asbestos fiber type, thorough fiber characterization, and careful dose-metric selection will continue to be critical determinants in evaluating disease outcomes, leading to important considerations in screening and risk assessment scenarios. More research should continue in susceptible populations such as pregnant women, children, and patients with underlying diseases. New research should also focus on the comparative toxicology and mode of action of asbestos fibers, as well as other hazardous EMPs such as erionite, winchite, antigorite, and more recently, nanomaterials. Additionally, research should include biomarkers of exposure (e.g., inflammasome-related molecules) and modalities for interfering with the mechanisms that lead to ARD (e.g., protein targets for autoantibodies and the inflammasome), which could reduce symptoms and asbestos-induced morbidity and mortality. Much of this research can also be used to support the mode of action of these various asbestos and EMP materials. Finally, it is only with a multidisciplinary approach that collective efforts will lead to an improved understanding of fiber-induced illnesses, new risk assessment strategies to describe potential risks, and new risk management approaches to help protect affected communities.

The authors declare they have no actual or potential competing financial interests. The research described in this article has been reviewed and approved for publication by the National Institute of Environmental Health Sciences, U.S. Environmental Protection Agency, and the Agency for Toxic Substances and Disease Registry. Approval does not signify that the contents necessarily reflect the views or the policies of the agencies or organizations, nor does mention of trade names or commercial products constitute endorsement or recommendation for use. D.J.C., T.C.L., J.C.P., S.H.G., A.S., A.M., and R.H. conceived, coordinated, and helped to draft the manuscript. All authors read and approved the final manuscript. The authors also wish to thank W. Suk and L. Birnbaum for their reviews of the manuscript.
